# Global colorectal cancer research, 2007‐2021: Outputs and funding

**DOI:** 10.1002/ijc.34279

**Published:** 2022-09-28

**Authors:** Mursheda Begum, Grant Lewison, Xiang Wang, Philip D. Dunne, Tim Maughan, Richard Sullivan, Mark Lawler

**Affiliations:** ^1^ Queen Mary University of London School of Business and Management London UK; ^2^ King's College London Institute of Cancer Policy, Guy's Hospital London UK; ^3^ Department of Medical Oncology Peking Union Medical College Hospital Beijing China; ^4^ Faculty of Medicine, Patrick G Johnston Centre for Cancer Research Health and Life Sciences, Queen's University Belfast Belfast UK; ^5^ MRC Oxford Institute for Radiation Oncology Gray Laboratories University of Oxford Oxford UK

**Keywords:** colorectal cancer, disease burden, funding, research domains, research outputs

## Abstract

The purpose of this study was to provide an evidence base for colorectal cancer research activity that might influence policy, mainly at the national level. Improvements in healthcare delivery have lengthened life expectancy, but within a situation of increased cancer incidence. The disease burden of CRC has risen significantly, particularly in Africa, Asia and Latin America. Research is key to its control and reduction, but few studies have delineated the volume and funding of global research on CRC. We identified research papers in the Web of Science (WoS) from 2007 to 2021, and determined the contributions of the leading countries, the research domains studied, and their sources of funding. We identified 62 716 papers, representing 5.7% of all cancer papers. This percentage was somewhat disproportionate to the disease burden (7.7% in 2015), especially in Eastern Europe. International collaboration increased over the time period in almost all countries except in China. Genetics, surgery and prognosis were the leading research domains. However, research on palliative care and quality‐of‐life in CRC was lacking. In Western Europe, the main funding source was the charity sector, particularly in the UK, but in most other countries government played the leading role, especially in China and the USA. There was little support from industry. Several Asian countries provided minimal contestable funding, which may have reduced the impact of their CRC research. Certain countries must perform more CRC research overall, especially in domains such as screening, palliative care and quality‐of‐life. The private‐non‐profit sector should be an alternative source of support.

## Graphical abstract

What's new?

Active research is key to high‐quality colorectal cancer (CRC) care. Here, the authors catalogued CRC research published between 2007 and 2021 with regard to study topics, funding, and the relative contribution of different countries. CRC accounted for 5.7% of all cancer papers, slightly less than its share of the disease burden. Some regions, such as Eastern Europe, are underperforming in CRC research relative to their economic status. Leading areas of research included genetics and surgery, and more research is needed on palliative care and quality‐of‐life. Some countries, including China and the US, provide substantial government funding for CRC research, while others rely on the charity sector. Overall, the private sector provided little funding.

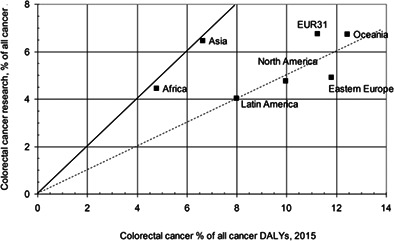

## 1 INTRODUCTION

Between 2000 and 2015, major improvements were achieved in global health, leading to increases in life expectancy.^1^, ^2^, ^3^ These improvements were particularly significant in low‐ and lower‐middle income countries (LMICs).^4^, ^5^ But as populations age, their cancer burden increases^6^ and cancers such as colorectal cancer (CRC) cause significant morbidity and mortality.^7^, ^8^ Thus, in 2000, CRC accounted for 0.51% of the world total disease burden, but in 2015 this had increased to 0.70%, according to the World Health Organization.^9^ The impact of CRC is measured in Disability Adjusted Life Years (DALYs), which take account both of early death and of time spent living with a disease. The increase in DALYs was 56% in Asia and in Latin America, and as much as 67% in Africa. CRC also increased its share of the overall cancer burden, with a rise of 9%, but with percentage increases of 27% in Latin America and 23% in Asia.^9^

Research is an essential aspect of high quality CRC care systems.^10^ Patients treated at research‐active hospitals have better outcomes than those who are not.^11^ However, to date there has been little systematic consideration of the state of global CRC research. The only publication that focused on the disease by itself was a list of the 100 most influential papers.^12^ However, this did not address the geographical spread of the total output, or its variation with time. No previous study has determined if CRC research was performed in the research domains most important for the understanding, control and treatment of the disease.

Some bibliometric studies of overall cancer research in selected geographical areas have shown that CRC is neglected by researchers relative to its burden of disease. As a proportion of all cancer research, CRC research appeared to be only 50% of what would be expected, based on the percentage of deaths in India in 2004.^13^ CRC research was also only about half the percentage, relative to CRC DALYs in Europe,^14^ although the situation improved between 2002‐2004 and 2011‐2013. However, in China, CRC may have been somewhat over‐researched in the period 2009 to 2018.^6^

In this article, we have identified peer‐reviewed published CRC research papers in the Web of Science (WoS, Clarivate Analytics) during the 12‐year period 2007 to 2018, and examined their characteristics, including their sources of funding (this data source was used because we have developed specialized software over several decades that enables us to process and analyze the data rapidly. We recently added papers for the last 3 years, 2019 to 2021, to bring the results up to date). We also looked at CRC clinical trials, given their relevance to improved clinical care. Our aims were: (i) to determine which countries were performing well, and which countries needed to do more research; (ii) to delineate the research domains that were well studied and by whom and (iii) to analyze the main financial sponsors of the research.

## 2 METHODOLOGY

Papers (articles, reviews) on cancer were identified in the WoS with a proprietary complex filter which contained the names of 185 specialist cancer journals and 323 title words or phrases, thus maximising the capture of CRC papers in both cancer and noncancer journals. The calibrated filter^15^ had a precision (specificity) of 0.95 and a recall (sensitivity) of 0.98. This meant that it over‐estimated the number of cancer papers by only 3% (details of the cancer research filter can be provided on request). To these cancer papers, an additional filter was also applied to identify the subset specifically on CRC at the intersection of the two filters. This second filter consisted of nine title words (*bowel*, *colon*, *colonic*, *colorectal*, *fap*, *lynch*, *polyposis*, *rectal* and *rectum*), and the names of four colorectal journals (*Colorectal Disease*, *Diseases of the Colon and Rectum*, *Inflammatory Bowel Diseases* and *International Journal of Colorectal Disease*). We also took all papers from the journal *Clinical Colorectal Cancer*. Bibliographic details of all the papers were downloaded for the 12 years 2007 to 2018 in March 2017 and April 2019 and converted into an MS Excel spreadsheet with a macro designed by Philip Roe (Evaluametrics Ltd). These included the author names and addresses, the paper title and source, and the text of the acknowledgement and list of funders. Subsequently (in April 2022) a further set of CRC papers from 2019 to 2021 were downloaded from the WoS so that any additional changes in output from the leading countries could be determined.

The fractional contribution of each country in the address field was calculated for each paper by means of another macro. For example, a paper with one UK address and two German addresses would be marked as UK = 0.33 and DE = 0.67. We focused on the leading 26 countries that had >150 papers published from 2014 to 2018 on a fractional count basis. These are listed in Table 1, with their International Organization for Standardization digraph (ISO2) codes. The outputs of the leading countries in 2014 to 2018 were plotted against their wealth (Gross Domestic Product, GDP, in 2015^16^). The time difference was designed to allow for the time taken for the research to be conducted and published. We grouped the countries by continent, and then compared these outputs relative to all cancer research with their collective burden in DALYs from CRC, and their overall cancer disease burden. This was performed in order to determine if CRC research was receiving sufficient attention, relative to that on other cancer anatomical sites. We also listed the leading individual organizations that published CRC research papers, and determined for each of them the corresponding total for all cancer research (ONCOL) and the ratio between them, which shows whether or not they placed additional focus on CRC research.

**TABLE 1:** List of leading 26 countries for colorectal cancer (CRC) research output (>150 papers in 2014‐2018), with their associated International Standards Organization digraph (ISO2) codes

Country	ISO2
Australia	AU
Austria	AT
Belgium	BE
Brazil	BR
Canada	CA
China	CN
Denmark	DK
Egypt	EG
France	FR
Germany	DE
Greece	GR
India	IN
Iran	IR
Israel	IL
Italy	IT
Japan	JP
Korea, South.	KR
Netherlands	NL
Norway	NO
Poland	PL
Spain	ES
Sweden	SE
Switzerland	CH
Turkey	TR
United Kingdom	UK
United States	US

A second macro was employed to characterize the papers by their research domain (such as genetics, surgery, quality‐of‐life), based on words in their title or the journals in which they were published. We determined the relative commitment of the leading countries to each type of research, in order to show which research domains predominated and also which might benefit from more international collaboration. We also subdivided the domain of genetics into four subject areas: heritability, sporadic CRC, genomic signatures and animal models.

Finally, we analyzed the funding sources for the papers published from 2009 to 2016. Since late 2008, the WoS has included explicit funding acknowledgement data as three searchable fields. However, it is also necessary to take account of implicit acknowledgements from paper addresses for government laboratories, those of commercial companies, and collecting charities (but not foundations). Because the names of financial sponsors are given in many different formats, we coded them with a three‐part code^17^ that included an identifier, the sector and the country. We took account of the numerous false positive inclusions of commercial firms (mainly pharmaceutical companies) in the listings of funding sources where they had been included in the acknowledgement text in order to declare a possible conflict of interest. We then used two further macros to analyze funding contributions. The first macro added the codes from the two thesauruses (acknowledgements and addresses) to each of the papers, while the second macro calculated the contributions of each funder to each paper, based on a double fractionation, and hence to the support provided from each funding sub‐sector to the CRC research of each country. The double fractionation took account of the proportion of addresses on each paper from each country, and of the numbers of funders from that country. For example, if France was one of three countries that had contributed to the paper, and there were two French national sources of funding (eg, one public and one PNP), then each of these was deemed to have contributed one‐sixth of the cost of the research.

## 3 RESULTS

**FIGURE 1:**
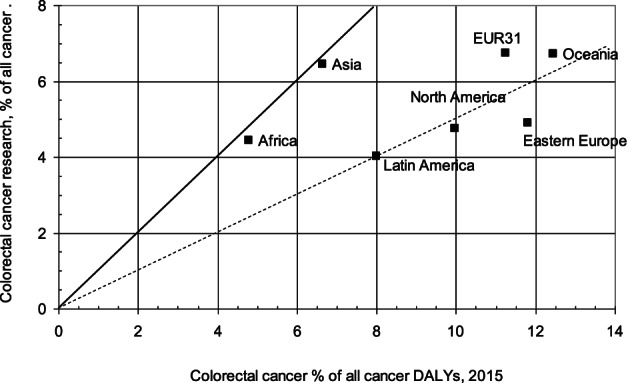
The percentage of all cancer research on colorectal cancer (CRC), 2014 to 2018, for seven continental regions, fractional counts of WoS papers, as a function of the percentage of the cancer disease burden in DALYs in 2015 (WHO data). Dashed line shows values half of those that would be proportionate

After the removal of 36 retracted papers, there were 62 716 papers in our database on CRC in the evaluable 12 year period, 2007 to 2018. The number doubled between 2007 and 2018, and the Annual Average Percentage Growth (AAPG) was 6.9%. The CRC papers represented 5.7% of the total publications for all cancers. This percentage rose slightly to 6.2% in 2014, but then dropped to 5.3% in 2016. However, it rose again to 6.3% in 2019 to 2021. CRC DALYs accounted for 7.7% of the cancer total in 2015, so research was less than proportionate by 31%. However, this shortfall varied greatly by geography, see Figure 1. Asia and Africa appear to be performing a proportionate amount of research, but the other geographical regions are publishing proportionally much less, particularly Eastern Europe (including Russia), where the relative disease burden is increasing. For this geographical region, there was virtually no correlation between the disease burdens from the different cancers and the amount of research performed on these cancers. Leukaemia was relatively over‐researched, as was skin cancer, but lung cancer was under‐researched by a factor of more than four, and CRC by a factor of more than two.

**FIGURE 2:**
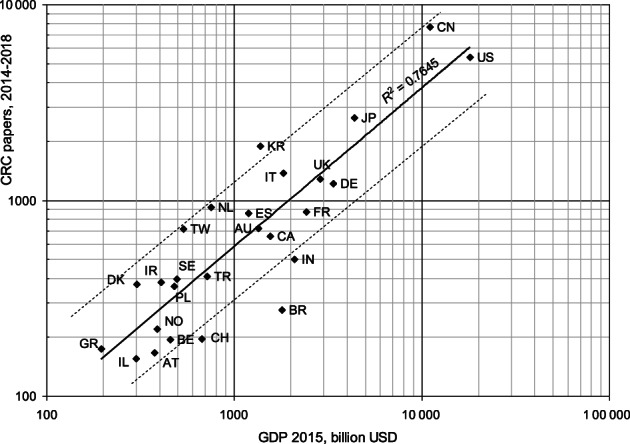
Outputs of CRC papers from individual countries in 2014 to 2018, fractional counts, compared with their Gross Domestic Products in 2015, US $ billion. Dashed lines show values that are twice and half those of the best least‐squares trend line. Country ISO2 codes listed in Table 1

Figure 2 shows a plot for the leading individual countries in which their fractional paper counts in the last 5 years are compared with their wealth (GDP) in 2015. This plot shows a reasonable correlation, but does not take account of the relative disease burden from CRC in the different countries. Some of the high‐performing countries had an increased CRC burden in 2015 compared with the world average burden of 0.7%, such as Denmark (DK, 2.8%), the Netherlands (NL, 2.7%) and South Korea (KR, 2.1%), so their concomitant higher research outputs were appropriate. However, although India (IN, 0.34%) experienced less than half the world mean CRC disease burden, Brazil (BR, 0.9%) and especially, Switzerland (CH, 2.0%) experienced above the average value for CRC DALYs, and appear to be neglecting CRC in their research portfolios. The countries varied greatly in their AAPG values: the value for China was 24%, but for the USA it was 2.3% and for Germany it was only 0.6%. As a result, China accounted for 28% of the world total in 2018, and its output overtook that of the USA in 2014.

The leading individual institutions, with more than 900 addresses during the period 2007 to 2018, are listed in Table 2. There is a relative paucity of institutions from China, because for much of this period its output was quite low. The mean ratio of COLON to ONCOL is 10.3%, which is higher than the mean presence of CRC within cancer research (5.7%,*v.s*.) because many CRC papers have repeated addresses from different departments within the same institution. For example, the total for Harvard University also includes papers from the Massachusetts General Hospital and the Brigham & Women's Hospital in Boston. This ratio is particularly high for Kaohsiung Medical University in Taiwan (24.6%) and the Leiden University Medical Centre in the Netherlands (23.5%), but low in the National Institutes of Health (5.0%) and the University of California system (6.3%) in the USA (overall, North America focuses relatively less on CRC than does Western Europe). However, the correlation between the two outputs is positive (*r* ^2^ = 0.49).

**TABLE 2:** The leading individual institutions in colorectal cancer research (COLON), 2007 to 2018, with their main cities (some institutions have multiple sites) and countries, and their estimated outputs in all cancer research (ONCOL), and the ratio between them

Institution	Location	COLON	ONCOL	Ratio
Harvard University	Cambridge MA USA	3153	28 097	0.112
UT MD Anderson Cancer Center	Houston TX USA	1859	25 724	0.072
Sun Yat Sen University	Guangzhou, China	1795	11 472	0.156
University of California System	USA	1732	27 422	0.063
INSERM	France	1517	17 813	0.085
Fudan University	Shanghai, China	1425	10 959	0.130
Memorial Sloan Kettering Cancer Ctr	New York NY USA	1407	15 506	0.091
Seoul National University	Seoul, S Korea	1318	9246	0.143
Shanghai Jiao Tong University	Shanghai, China	1248	10 911	0.114
German Cancer Research Ctr DKFZ	Heidelberg, Germany	1175	7409	0.159
Mayo Clinic	Rochester MN USA	1172	13 480	0.087
Kaohsiung Medical University	Kaohsiung, Taiwan	1166	4749	0.246
Zhejiang University	Hangzhou, China	1092	7442	0.147
Yonsei University	Seoul, S Korea	1068	6033	0.177
Leiden University	Leiden, Netherlands	1049	4465	0.235
Johns Hopkins University	Baltimore MD USA	1040	13 756	0.076
University of Toronto	Toronto ON Canada	1022	11 414	0.090
Natl Inst Hlth & Natl Canc Inst	Bethesda MD USA	941	18 702	0.050
			*CORREL*	0.70
			*R2*	0.49

*Note*: the number of addresses found in COLON may exceed the number of papers.

The partition of the papers by their research domain for the 12 year period shows that the three leading domains were genetics (21%), surgery and prognosis (both 18%). Within genetics, the two largest areas were sporadic CRC (2598 papers, 19% of genetics) and inherited CRCs (2136 papers, 15%). Outputs of genomic signatures and of animal model papers were much smaller (437 and 388, respectively). Relative to the world, the USA over‐performed in inherited genetics and models by 40%, while China under‐performed in these two subfields by 71% and 38%, respectively. As a result, these subfields both decreased their shares of the world output in the genetics domain over the study period, especially in inherited genetics.

Although surgery accounted for 18% of all CRC research world‐wide, it was only 10% in Europe, 8% in India, and 7% in China.^6^, ^13^, ^14^ This focus on surgical research reflects its primacy as the major treatment option for CRC. During the last 5 years, research on disease prognosis (PROG) overtook surgery, with 6623 papers (20% of the total in those years). There is an unusually small amount of research on palliative care (0.5%), which is almost universally neglected as a cancer research domain. In the last 3 years (2019‐2021), prognosis increased its share to 22% of the total, but surgery declined to 18%. Palliative care also increased, to 1.0% of the total in these 3 years.

There were 2373 papers describing clinical trials (3.6% of the total in 2007‐2018). The countries with the relatively greatest number were Belgium (14% of its papers), Austria (8.2%), France (6.7%), Norway (6.4%) and Japan (5.4%). Of the 327 Japanese clinical trials papers, 53% were on chemotherapy and 26% on surgery. Other Asian countries, however, did relatively few clinical trials as judged by their publication output.

The values of relative commitment to different research domains by the leading countries are shown in Table 3. Surgery, the main treatment modality, is researched preferentially in Japan (JP; ×2.1), South Korea (KR; ×1.5) and several European countries, but proportionately less in Iran (IR; ×0.2), India (IN; ×0.5) and China (CN; ×0.5). Radiotherapy is researched at an above‐average level in South Korea (×2.0), the Netherlands (NL; ×1.7) and Belgium (BE; ×2.2). Chemotherapy (both conventional and targeted therapy) is researched relatively more highly in Japan (×1.8) and France (FR; ×1.6). Screening is actively researched only in the US (×2.5), the UK (×1.9), Canada (CA) and Spain (ES; ×1.8), and Australia (AU; ×1.7). It appears to be neglected in most other countries, particularly in Asia (all these ratios are statistically significant at *P* < 0.5%, except for Belgium's commitment to radiotherapy research, for which *P* > 5%).

**TABLE 3:** The relative concentration on 12 research domains within CRC research, 2014–18, for 26 leading countries

Ratio	*GENE*	*SURG*	*PROG*	*EPID*	*PATH*	*CHEM*	*RADI*	*SCRE*	*DIAG*	*TARG*	*QUAL*	*PALL*
CN	**1.27**	**0.51**	1.05	**0.75**	**1.17**	0.91	**0.67**	**0.22**	0.86	**0.61**	**0.25**	**0.31**
US	**0.91**	**0.91**	**0.86**	**1.56**	**0.80**	**0.70**	**0.84**	**2.47**	*1.07*	**0.77**	*1.24*	1.45
JP	**0.73**	**2.05**	1.10	**0.65**	*1.01*	**1.65**	*1.14*	**0.23**	*0.86*	**2.03**	**0.54**	*1.00*
KR	0.91	**1.49**	1.14	*1.16*	*1.00*	0.87	**1.95**	**0.60**	**0.65**	**0.55**	**0.50**	*1.03*
IT	*0.89*	**1.19**	0.89	**0.70**	*0.85*	*1.25*	1.28	**0.68**	*1.07*	**2.21**	*0.80*	0.43
UK	**0.60**	**1.32**	**1.26**	*1.19*	*1.03*	*0.86*	*1.19*	**1.87**	**1.77**	*0.77*	**2.07**	*1.31*
DE	*1.02*	1.13	**1.18**	*0.86*	1.24	*0.89*	*1.06*	*1.15*	*0.98*	*1.13*	*0.84*	0.42
NL	**0.75**	**1.49**	*1.11*	1.27	**0.70**	*1.21*	**1.73**	1.37	*1.13*	*0.85*	**4.64**	2.91
FR	**0.75**	**1.33**	*0.92*	*0.83*	*0.83*	**1.53**	*1.12*	*1.14*	*1.11*	**2.05**	**1.41**	*1.16*
ES	*0.95*	*0.96*	*1.04*	*0.93*	**0.68**	*1.00*	*0.93*	**1.77**	1.46	1.46	*0.79*	0.62
AU	*1.01*	*0.89*	*1.08*	*1.23*	*0.98*	*1.03*	*1.31*	**1.69**	*0.86*	*1.43*	2.10	2.55
CA	*0.80*	*1.08*	*0.97*	1.42	0.71	**1.62**	*1.26*	**1.84**	*1.11*	*0.90*	2.01	*1.98*
IN	0.84	**0.53**	**0.40**	**0.34**	0.79	*1.18*	*1.01*	**0.18**	*0.86*	**0.41**	**0.17**	**0.04**
TR	*0.89*	*1.15*	*1.12*	*0.88*	*1.35*	*0.83*	*0.92*	**0.53**	*0.79*	1.89	*0.85*	*1.22*
SE	*0.97*	**1.42**	*1.11*	**1.65**	*0.95*	*0.80*	*0.83*	*0.64*	*0.83*	0.49	3.05	*3.09*
IR	**1.46**	**0.20**	**0.66**	*0.91*	*0.88*	*1.15*	*0.72*	*0.66*	*1.27*	0.51	*1.22*	*1.89*
DK	*0.98*	1.24	*1.10*	**1.75**	*1.12*	*0.99*	*0.91*	*1.29*	*1.50*	*0.99*	*1.72*	*1.17*
PL	1.28	*0.86*	**0.69**	*0.76*	*1.38*	*0.79*	*0.81*	**0.36**	*1.15*	0.52	*1.10*	*1.37*
BR	1.32	*0.83*	*0.85*	0.57	*1.15*	*1.15*	*1.49*	0.34	*0.81*	*0.62*	*2.30*	*3.01*
NO	*0.97*	*1.18*	**1.64**	*1.40*	*0.78*	*0.98*	*1.52*	*1.31*	0.45	**0.35**	*0.82*	*1.47*
CH	*0.97*	*1.10*	1.33	*0.68*	*1.50*	*0.95*	*1.19*	*0.77*	*2.05*	*1.17*	*0.44*	*0.85*
BE	*0.70*	*1.28*	*1.22*	**0.46**	*0.64*	*1.26*	2.20	*0.53*	*0.68*	*2.62*	*0.41*	**0.09**
GR	1.29	*0.95*	*1.17*	0.56	*1.16*	*1.06*	**0.06**	*0.67*	*1.22*	*1.52*	*0.50*	*1.02*
AT	*1.17*	*1.05*	1.41	*0.66*	1.73	**1.93**	*0.91*	*0.61*	*0.64*	*1.81*	*0.69*	*0.10*
IL	*0.92*	*1.08*	**0.52**	*1.17*	*0.69*	*0.77*	*0.97*	*1.35*	*1.48*	*1.01*	*0.00*	*1.07*
IE	*0.64*	*1.20*	*1.10*	*0.91*	*1.17*	*0.88*	*1.29*	*1.75*	2.81	*0.43*	*4.32*	*2.30*

*Note*: For ISO2 codes, see Table 1. For domain codes, see caption to Figure 3. Values that differ from unity with statistical significance *P* < 0.5% shown in bold type; values for *P* < 5% shown in roman type; values not statistically significant at *P* < 5% shown in italics.

Those countries that are in relative terms neglecting certain research domains might benefit from additional international collaboration to balance their research portfolios. International collaboration varied greatly between countries, but has mostly been increasing with time. Although the percentage of foreign contributions to Chinese papers has declined between 2007‐2010 and 2015‐2018 (from 12% to 7%), the actual number of these contributions has risen from 32 per year to over 122 per year between the first and last quadrennia. The UK (+76%) and the USA (+64%) have increased their international collaboration the most over the study period. It is notable that the three most productive Asian countries (China, Japan and South Korea) collaborate much less than CRC researchers in Europe or North America, probably because of language and geography. This is also true for India (international papers = 27%), Iran (20%) and especially Turkey (10%).

**FIGURE 3:**
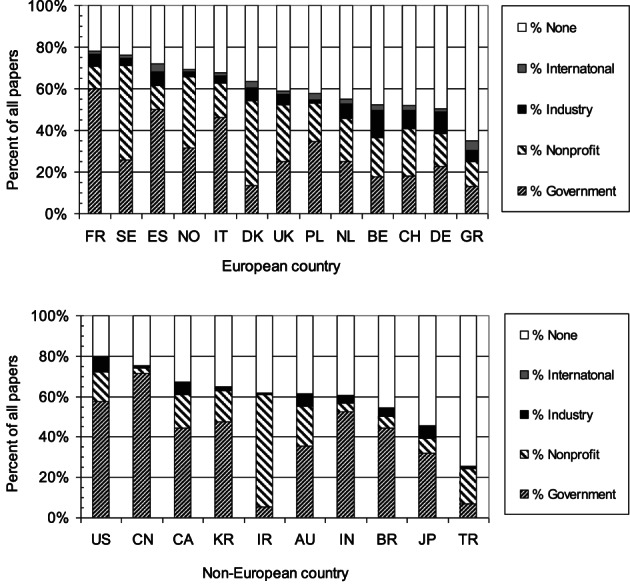
Sources of funding for 13 European country (upper chart) and 10 non‐European country CRC papers (lower chart), 2007 to 2016, by main sector. Countries ranked by percentages of papers with funding. ISO2 codes in Table 1

Funding sources for the CRC research papers are shown in two charts, one for 13 European countries (Figure 3, top) and one for 10 non‐European countries (Figure 3, bottom). For most European countries, the support from the private‐non‐profit (PNP) sector is greater than that from government, especially in the Scandinavian countries (DK, NO and SE), and in Belgium (BE), Switzerland (CH) and the UK. The second chart shows mainly the reverse, with government funding predominating, except for Iran (IR) and Turkey (TR). However, the composition of the PNP sector varies greatly, and the percentage shares are shown in Table 4. Collecting charities comprised just under one third of the total, but much more in the UK (67%) and the Netherlands (NL; 64%) reflecting the significant contribution to CRC research by Cancer Research UK and the Dutch Cancer Society. However, the funding contribution of cancer charities was marginal outwith Europe, except in Australia (AU; 57%) and Canada (CA; 53%), and to a lesser extent in the USA (27%). In the other countries, the largest source of PNP support was the universities' own funds, especially in Turkey (TR; 91%) and Iran (IR; 90%). This means that researchers received largely noncontestable research funding, with an inevitable lack of clarity in relation to peer review. Endowed foundations were notable in Denmark, where there are many small foundations, named for successful men and their wives (see Table 4),^17^ and a few large ones, such as Lundbeck and Novo Nordisk.

**TABLE 4:** The funding sub‐sectors contributing to the total government and private‐non‐profit sectoral contributions to CRC research in 23 leading countries, 2007 to 2016

	Government			Private‐non‐profit				
ISO2	DEPT	AGENCY	LA	CHAR	FDN	HOSP	UNIV	OTH
AU	8.1	56	36	57	2.9	9.7	21	9.4
BE	48	6.4	45	51	0.8	23	23	1.6
BR	4.9	50	45	14	0	22	58	5.6
CA	3.0	36	61	53	2.1	9.5	26	10
CH	17	77	5.9	53	23	8.5	8.4	7.0
CN	23	31	47	1.1	4.5	1.5	87	5.5
DE	27	61	12	30	24	4.1	26	15
DK	58	19	22	30	45	14	7.6	3.5
ES	17	48	35	27	22	8.7	13	29
FR	4.7	90	5.4	60	0.8	9.1	7.5	23
GR	72	28	0	2.5	14	4.6	52	26
IN	41	54	4.8	4.5	12	9.3	72	2.6
IR	98	0	2.0	0	0	3.7	90	6.5
IT	29	58	13	52	16	3.5	24	4.5
JP	76	23	0.6	17	27	7.1	25	24
KR	93	5.9	0.8	0.1	9.8	22	65	2.6
NL	27	72	0	64	8.5	10	7.5	9.5
NO	4	51	45	45	7.9	28	12	7.7
PL	67	31	2.5	1.7	0	0	86	12
SE	0.1	30	70	38	23	9.3	19	10
TR	100	0	0	0	0	1.0	91	7.8
UK	67	33	0.2	67	7.0	7.1	11	7.7
US	10	86	3.3	27	15	9.5	33	15
Mean	29	36	27	33	13	11	32	11

*Note*: Figures are percentages of total government and PNP support. ISO2 codes for countries in Table 1.

Abbreviations: AGENCY, government agency; CHAR = collecting charities; DEPT, government department; FDN, endowed foundations; HOSP, hospital own funds; LA, local or regional government; OTH, other nonprofit (eg, professional associations); UNIV, university own funds.

There was also a big variation in the governmental sector, between departments (under direct ministerial control) and agencies (intended to be independent). In some countries, notably Sweden (SE; 70%), Canada (CA; 61%), China (CN; 47%), Belgium (BE; 46%), Brazil (BR; 45%) and Norway (NO; 45%), local or regional authorities were substantial funders of this research (Table 4). The percentage contribution of government agencies, which usually have a contestable grant funding system with peer review, is particularly strong in France (FR; 90%), the USA (86%) and Switzerland (CH; 77%).

Industry funded only 4.9% of the research. This is much less than for lung cancer, which was reaching 8% in 2009 to 2013.^18^ Figure 3 shows that commercial funding is not a major contributor to CRC research, except in Belgium (BE; 13%) and Germany (DE; 10%). Overall, pharma companies contributed 46% of the total, and biotech companies, 15% (although the distinction between them is often not clear), and nonpharma companies, 40%. The latter is an unusually high percentage, and may reflect the research emphasis on surgery, and its requirements for imaging and mechanical equipment, rather than chemotherapy. The leading pharma company was the Swiss company F Hoffman La Roche s.a., who provided support equivalent to that estimated for 181 papers. It was followed by Sanofi‐Aventis (France, 82 papers), Pfizer (US, 65 papers), Merck KGaA (Germany, 64 papers), Bayer (Germany, 54 papers), Taiho Pharma (Japan, 51 papers) and AstraZeneca (UK) and Merck Inc. (US, both 38 papers). The leading nonpharma companies (Olympus in the USA, Covidien in Ireland and Philips nv in the Netherlands) provided much less support, equivalent to 21, 14 and 13 papers, respectively.

Finally, the international funding sector (primarily the European Union [EU] with its many research funding programs) funded the equivalent of 312 papers, 0.8% of the world total.

## 4 DISCUSSION

Research is an important component of a country's performance in the provision of evidence to underpin improvement of the care of its cancer patients. Because of the rising global burden of CRC, clinicians, scientists, government and state policy‐makers have championed cancer research, from prevention, screening and diagnosis through to patient treatment and palliation. However, the amount of CRC research being performed is still low relative to the burden caused by the disease, especially in some world regions such as Eastern Europe. The quality of the research must also be considered.

The needs of CRC research in the individual countries will vary, because there are substantial differences in the impact of the disease.^19^ Our study reveals that certain countries, especially in Eastern Europe, are underperforming in CRC research relative to their GDP and need to do more to address the rising burden of this cancer. Countries such as Brazil and Switzerland may also need to change their research portfolios, to respond to the specific CRC‐related challenges they face.

Recent technological advances in CRC diagnosis have underpinned enhanced clinical research activity. Because of improvements in genomic technologies, molecular profiling has become cheaper and more accessible for cancer researchers and clinical investigators. This has furthered our understanding of the molecular behavior of CRC.^20^ Moreover, knowledge of the associated clinical ramifications of molecular subtypes of CRC can help optimise treatment strategies and predict patient outcomes.^21^, ^22^, ^23^ It is therefore not surprising that genetics/genomics is the most popular research domain, according to our study. But the research perhaps needs to be more proportionate to the needs of individual countries or regions, particularly in Central and Eastern Europe.

Broad technological advances have also been made in CRC therapy, including in surgery, radiotherapy^24^ and molecular‐based treatment.^21^, ^24^, ^25^, ^26^ These are therefore significant research domains. In particular, thanks to improvements in surgical techniques,^25^, ^27^, ^28^ increasing attention has been paid to this research domain, due to its primacy in the improvement of survival. However, there are significant regional variations, because of a lack of definitive research studies^22^ and this is very much reflected in our data.

Although CRC morbidity and mortality can be mitigated through appropriate screening and surveillance approaches, these research areas appear to be neglected in many countries, especially in Asia. This may be due to a shortage of human and financial resources, but also a lack of awareness of the need for these types of research. Tumor heterogeneity in CRC has been identified, and many approaches have been developed to determine patient prognosis based on the biology of individual tumors and personal characteristics.^29^ Thus, research on the prognosis of CRC has attracted more and more attention, and its output recently overtook that in genetics. Palliative care research has been severely neglected, perhaps because it requires more collaboration from workers from several different disciplines who provide support to patients. However, recently it has received more attention, although it still represents barely 1% of the total research output.

Alternative sources of funding are evidently needed and should be actively sought by governments and societies. This will involve fiscal encouragement to the charitable sector, and the formation and support of medical research charities in geographical areas where they do not currently exist. However, this is particularly difficult in LMICs, where medical research and science are usually low on the list of national priorities. In this context, international collaboration can be an important source of additional funding, and can also provide a level of peer review for national funding bodies. For example, in the Czech Republic international collaborative proposals that are deemed fundable by say an EU funding scheme, but fall just below the overall budget threshold, may be funded at national level following the positive peer review.

The study has some limitations. We used a single database of research outputs (the WoS). This has some language biases, omitting a proportion of clinical papers in national languages, especially from East Asia. For example, the papers from China are in much more basic/discovery research on average than those from the rest of the world, so it is likely that a number of clinical papers will have been omitted from the WoS. A few funding sources could not be coded, as no information about them was available on the Web, or they were given in the acknowledgements only as initials. There were also some funding references where their name was not given, only the grant number, and not all of these sources could be identified. Nonetheless, the data accumulated and evaluated in this study provide crucial intelligence to help guide our collective research efforts to understand CRC and deliver research‐informed insights that will help reduce its global burden.

## AUTHOR CONTRIBUTIONS

Conceptualization: Richard Sullivan, Mark Lawler. Data curation: Mursheda Begum. Formal Analysis: Mursheda Begum, Grant Lewison. Funding acquisition: Tim Maughan, Mark Lawler. Investigation: Mursheda Begum, Grant Lewison. Methodology: Grant Lewison. Project administration: Grant Lewison. Resources: Richard Sullivan. Software: Philip Roe, Evaluametrics Ltd. Supervision: Grant Lewison. Validation: Xiang Wang, Philip D. Dunne, Tim Maughan, Richard Sullivan, Mark Lawler. Visualization: Grant Lewison. Writing—original draft: Grant Lewison, Mark Lawler, Richard Sullivan. Writing—review & editing: All authors. The work reported in the paper has been performed by the authors, unless clearly specified in the text.

## FUNDING INFORMATION

This study was supported by a Medical Research Council—Cancer Research UK (MRC—CRUK) Stratified Medicine in Colorectal Cancer (S:CORT) Program Grant and a grant from Health Data Research UK.

## CONFLICT OF INTEREST

Mark Lawler and Richard Sullivan are in receipt of an unrestricted educational grant from Pfizer for research unrelated to this work. Mark Lawler has received honoraria for speaking engagements from Pfizer, EMF Serono, Novartis, Bayer and Roche for research unrelated to this work. Tim Maughan has received consultancy fees from AstraZeneca and Pierre‐Fabre unrelated to this work. The other authors declare no conflict of interest.

**Abbreviations**
CRC: colorectal cancer
DALY: disability‐adjusted life year
GDP: gross domestic product
ISO: International Organization for Standardization
LMIC: lower middle income country
MS: Microsoft Inc.
nv: Naamloze Vennootschap (Dutch limited company)
PNP: private nonprofit
s.a.: Société Anonyme (French or Swiss limited company)
WoS: Web of Science (Clarivate Analytics)

## Data Availability

The data that support the findings of this study are available from the corresponding author upon reasonable request.
